# Synergistic SNGI-TKI combination against LSCs

**DOI:** 10.18632/aging.101272

**Published:** 2017-07-30

**Authors:** Xiaoyan Jiang

**Affiliations:** Terry Fox Laboratory, British Columbia Cancer Agency and Department of Medical Genetics, University of British Columbia; Vancouver, BC, Canada

**Keywords:** cancer stem cells, Icaritin (SNG162 and SNG1153), drug-resistance, tyrosine kinase inhibitors, CML, leukemic stem cells, BCR-ABL-T315I mutation

Overcoming drug resistance and targeting cancer stem cells (CSCs) remain major challenges for treatment of various cancers. Protein tyrosine kinases have been identified as major therapeutic targets in cancer and several tyrosine kinase inhibitors (TKIs) have been successfully used as molecular targeted therapies in cancer treatment. In particular, the TKI imatinib mesylate (IM) and new generations of TKIs, which specifically target the kinase activity of BCR-ABL in chronic myeloid leukemia (CML), have transformed CML from a fatal disease to a manageable disease [1]. Although these TKIs have demonstrated remarkable clinical efficacy against chronic phase (CP) CML, TKI monotherapy is not curative; reduced efficacy of TKIs in treating accelerated phase (AP) and blast crisis (BC) CML, coupled with the development of primary and acquired resistance to these compounds, remain significant challenges [2]. Relapses are frequently associated with point mutations in the BCR-ABL TK domain, with more than 100 mutations documented. In particular, most TKIs cannot target a critical T315I gatekeeper mutation in BCR-ABL that is found in TKI-resistant patients [2]. Importantly, TKI monotherapy is much less effective in eradicating leukemic stem cells (LSCs), the key cell population that generates minimal residual disease and drives disease relapse [3]. These observations emphasize the need to develop new therapeutic agents and combination strategies to specifically target LSCs and BCR-ABL-T315I mutant cells.

It has been reported that a small molecular inhibitor (Icaritin, SNG162), a key component of Epimedium flavonoid isolated from Epimedium Genus, inhibits growth of breast cancer cells by targeting abnormal activity of human estrogen receptor alpha 36 (ERα36), an alternative splicing variant of human estrogen receptor alpha 66 (ERα66). Interestingly, recent evidence demonstrates that it has much broader anti-cancer activity in many cancer types, including lung cancer, multiple myeloma, hepatocellular carcinoma, and human leukemia [4]. Indeed, we have recently demonstrated for the first time that ERα36 is highly expressed in BCR-ABL^+^ leukemic cells, including BCR-ABL-T315I mutant cells, and abnormally localizes to the cytoplasm and cell membrane of these cells, differing from full-length ERα66 [5]. More interestingly, increased expression of surface ERα36 was detected in primitive CD34^+^ IM-nonresponder cells compared to IM-responder cells from CML patients, and knockdown of ERα36 in CML cells increased the sensitivity of these cells to IM treatment, while suppression of full length ERα66 did not have an inhibitory effect. These results provide new insights into the critical role of ERα36 in mediation of TKI response/resistance of primitive leukemic cells.

Mechanistically, Icaritin (SNG162) and a newly developed analog of Icaritin (SNG1153), with enhanced potency and specificity, have been demonstrated to target key signaling proteins and regulate multiple cell signaling and survival pathways in different types of cancer cells. For example, it has been reported that SNG1153 inhibits growth and induces apoptosis in lung cancer cells, including CSCs, by disrupting WNT/β-catenin signaling through induction of β-catenin phosphorylation and protein degradation in these cells [6]. SNG inhibitors (SNGI) also inhibit multiple myeloma and hepatocellular carcinoma initiation by inactivating the IL-6/JAK2/STAT3 pathway [7]. In addition, it has been suggested that these compounds target cancer cells through additional mechanisms such as cell cycle modulation, anti-angiogenesis, anti-metastasis and immunomodulation [4]. Recently, we provide strong evidence that SNG162 and SNG1153 inhibitors, especially more potent SNG1153, effectively target IM-resistant BCR-ABL^+^ blast cells and BCR-ABL-T315I mutant cells [5]. This occurs through a distinct molecular action, which inhibits phospho-rylation of the Tyr177 residue at the regulatory domains on BCR and prevents its binding to a key regulator GRB2, leading to reduced activities of the downstream RAS-MAPK pathway. Specifically, we examined whether SNG inhibitors plus a TKI, to dually inhibit the BCR-ABLTyr177-mediated GRB2-RAS-MAPK path-way in addition to BCR-ABL activity, might be a promising treatment for CML patients unlikely to respond to TKI monotherapies, as it could more effectively reduce the CML stem cell burden, and prevent the development of TKI-resistance and disease relapse. The study on CD34^+^ treatment-naive IM-nonresponder cells provides direct support for this hypothesis. SNG inhibitors, in combination with a TKI, markedly reduced the output of progenitor colonies and their more primitive stem cells *in vitro*, while these concentrations were much less toxic to primitive healthy bone marrow cells. Oral SNG1153 inhibitor, combined with a TKI, effectively eliminates infiltrated blast cells and significantly enhances survival of mice. These results provide strong scientific rationale for a therapeutic combination strategy to specifically target TKI-insensitive stem/progenitor cells and mutant cells.

Encouragingly, SNG inhibitor phase I/II clinical trials in China have recently been completed, with encouraging results for patients with advanced hepato-cellular carcinoma and advanced solid tumors (NCT01278810, NCT01972672, NCT02496949). A phase III trial will be initiated soon. Thus, the availability of clinically safe SNG inhibitors provides opportunities to exploit improved treatment strategies for cancer therapy.
